# Diversification of Antitumour Immunity in a Patient with Metastatic Melanoma Treated with Ipilimumab and an IDO-Silenced Dendritic Cell Vaccine

**DOI:** 10.1155/2016/9639585

**Published:** 2016-07-18

**Authors:** Mouldy Sioud, Marta Nyakas, Stein Sæbøe-Larssen, Anne Mobergslien, Steinar Aamdal, Gunnar Kvalheim

**Affiliations:** ^1^Department of Immunology, Oslo University Hospital, Montebello, 0310 Oslo, Norway; ^2^Department of Clinical Cancer Research, Oslo University Hospital, Montebello, 0310 Oslo, Norway; ^3^Department of Cell Therapy, Oslo University Hospital, Montebello, 0310 Oslo, Norway

## Abstract

Indoleamine 2,3-dioxygenase (IDO) expression in dendritic cells (DCs) inhibits T-cell activation and promotes T-cell differentiation into regulatory T-cells. Moreover, IDO expression promotes resistance to immunotherapies targeting immune checkpoints such as the cytotoxic T lymphocyte antigen-4 (CTLA-4). Here, a patient with metastatic melanoma pretreated with ipilimumab, an anti-CTLA-4 blocking antibody, was vaccinated with IDO-silenced DCs cotransfected with mRNA for survivin or hTERT tumour antigens. During vaccination, T-cell responses to survivin and hTERT tumour antigens were generated, and a certain degree of clinical benefit was achieved, with a significant reduction in lung, liver, and skin metastases, along with a better performance status. T-cell responses against MART-1 and NY-ESO-1 tumour antigens were also detected in the peripheral blood. The patient also mounted an antibody response to several melanoma proteins, indicating diversification of the antitumour immunity in this patient. The identification of such serum antibody-reacting proteins could facilitate the discovery of tumour neoantigens.

## 1. Introduction

Dendritic cells (DCs) are professional antigen-presenting cells that bridge innate and adaptive immunity [1]. Given their potent role in activating cytotoxic T lymphocytes, DCs are being developed as vaccines for the treatment of patients with cancer [2]. However, despite huge effort to establish DC vaccination as a treatment for cancer patients, therapeutic success has been limited thus far [3, 4]. It has become quite evident that one key reason for the unsatisfactory clinical results is the presence of immune suppressive elements in the immune system and tumour microenvironment [4].

Among the factors contributing to immunosuppression is the population of regulatory cells, including T-regulatory (Treg) cells, tolerogenic DCs, and myeloid-derived suppressor cells (MDSCs) [3–6]. Treg cells and MDSCs can communicate with other cells such as DCs to suppress their immune-activating function [7]. One mechanism used by tolerogenic DCs involves the expression of immune inhibitory factors such as interleukin-10 (IL-10), indoleamine 2,3-dioxygenase (IDO), and programmed death ligand-1 [8, 9]. IL-10-induced immunosuppression is mediated by the inhibition of DC maturation and T-cell and NK-cell functions [10]. IDO-positive DCs suppress T-cell responses and promote tolerance via the depletion of tryptophan and the production of metabolic products, which in turn can induce a general immunosuppressive effect [8, 11]. Moreover, IDO-positive DCs induce T-cell differentiation into Treg cells instead of undergoing clonal expansion and differentiation into effector cells [8, 12, 13]. In the case of cancer vaccines, IDO expression can occur during in vitro production of DCs from CD14+ monocytes, as well as in vivo after T-cell activation [8, 14]. Agents required to differentiate monocytes into DCs ex vivo, such as prostaglandin E2 and Toll-like receptor ligands (e.g., R848), induce the expression of immunosuppressive factors, thus limiting the efficacy of today's cancer vaccines [15]. Given the immunosuppressive role of IDO, we speculated that its inhibition by RNA interference in DCs may enhance the potency of cancer vaccines [14]. As a proof of concept, we recently evaluated the safety and efficacy of an IDO-silenced DC vaccine in four patients with ovarian cancer and metastatic tumours [16]. The data indicated that the vaccine is safe and the immune responses observed in the patients were translated into objective clinical responses. In the present report, the immune and clinical responses to an IDO-silenced DC vaccine were investigated in a patient with advanced melanoma pretreated with ipilimumab, a monoclonal IgG1 anti-CTLA-4 neutralizing antibody [17]. The expression of CTLA-4 on activated T lymphocytes counteracts positive stimulatory signals to these cells mediated through costimulatory molecules expressed by antigen-presenting cells such as DCs.

## 2. Presentation

In 2006, a 14-year-old male underwent surgery for a benign nevus on the right side of the dorsum. The tumour was not examined since it was regarded as benign. In January 2009, the patient was diagnosed with a malignant melanoma in the same area. Following surgery of the primary tumour, a few months later the patient developed metastasis in the right axillary lymph nodes. In July 2009, a lymphadenectomy was performed and 1 of the 18 lymph nodes examined was positive. In June 2010, the patient developed recurrent disease in the right axilla, which was surgically resected. In January 2011, a new tumour localized to the same area was also surgically resected. In September 2011, lung metastasis developed along with a skin relapse in the site of the previously resected tumour on the dorsum. The skin lesions were surgically resected and the lung metastasis was treated with radiotherapy (15 GY × 3 treatment every 2nd day). In January 2012, the number of lung metastases increased and new metastasis appeared in the surgically affected axilla. In May 2012, therapy was initiated with ipilimumab (3 mg/kg given every 3rd week). After the second cycle of treatment, the patient developed severe side-effects and the treatment was discontinued. In July 2012, progressive disease developed in the lung, skin, and axilla. In early August 2012, palliative radiotherapy against the metastasis in the axillary lymph nodes was administered (3 GY × 10). However, the disease further developed and, in late August, metastasis was present in the lung, liver, bone, and lymph nodes. The patient received palliative radiotherapy against bone metastasis in the left knee (3 GY × 10). The liver metastasis was also treated with radiotherapy. In October 2012, radiotherapy against a brain metastasis was administered (25 GY × 1). Given the rapid disease progression, on November 16, 2012, the patient received an autologous IDO-silenced DC cancer vaccine as compassionate use. The patient received a combination of hTERT (right arm) and survivin (left arm) DC vaccines cotransfected with IDO siRNA (5 × 10^6^ DCs/vaccination). Intradermal revaccination occurred weekly for 4 weeks after the first DC injection. Booster vaccinations were administered every 4 weeks as a follow-up treatment until June 6, 2013.

### 2.1. In Vitro Characterisation of the DC Cancer Vaccine

**Figure 1:**
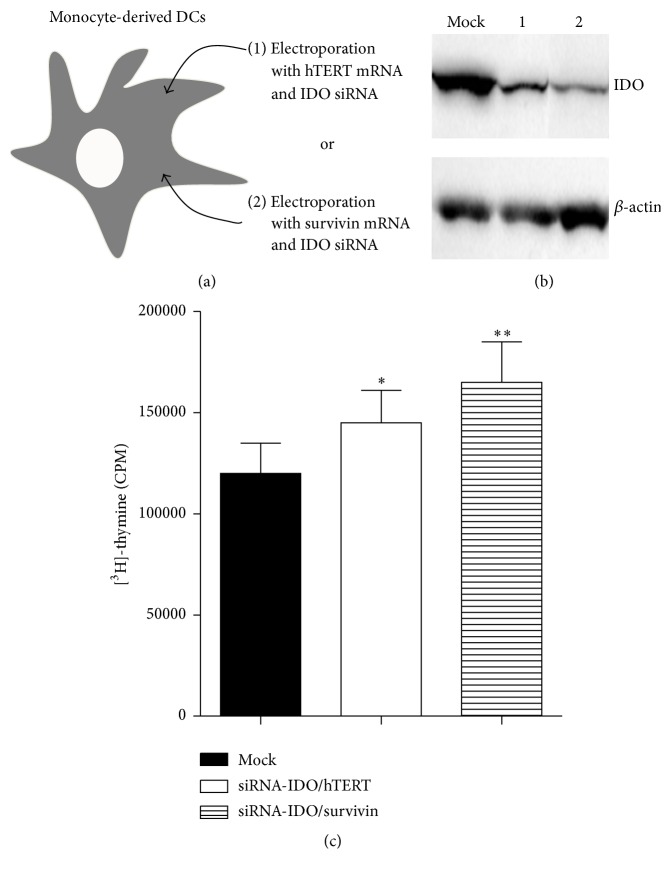
IDO-silenced DC vaccine. (a) Schematic illustration of the vaccine design. (b) IDO gene silencing in the DC vaccine. Fast DC preparations were transfected with IDO siRNA under GMP conditions, as described previously [14]. At 24 h after transfection, IDO expression was investigated by western blots, and the remaining cells were cryopreserved into vaccine doses and stored in a GMP-dedicated area. Lanes 1 and 2 correspond to DCs transfected with IDO siRNA and mRNA encoding for hTERT and DCs transfected with IDO siRNA and mRNA encoding for survivin, respectively. Mock DCs were not transfected with IDO siRNA. (c) Potency of IDO-silenced DC in stimulating allogeneic T-cells. Patient IDO-silenced DCs and IDO-positive DC vaccine preparations were cocultured with CD4+ T-cells from a healthy donor at a T-cell DC ratio of 1 : 5. After 5 days in culture, T-cell proliferation was determined by incorporation of [^3^H]-thymidine following overnight pulsing. The results are presented as the mean ± standard deviation of triplicate determinations. ^*∗*^ *P* < 0.05 and ^*∗∗*^ *P* < 0.02. IDO: indoleamine 2,3-dioxygenase; siRNA: small interfering RNA; DC: dendritic cells.

Specific gene silencing of IDO in DC vaccines was confirmed by western blot analysis (Figures 1(a) and 1(b)). The expression of the HLA-DR and costimulatory molecules, including CD86, CD80, and CD40, was not affected by the IDO gene silencing when compared with the untransfected DCs (data not shown). When compared with the mock-transfected DCs, IDO-silenced DCs enhanced the activation of allogeneic T-cells (Figure 1(c)), suggesting that IDO protein is functional in DC preparations and its inhibition releases T-cell activation (*P* < 0.05).

### 2.2. Immune Response to Vaccination

**Figure 2:**
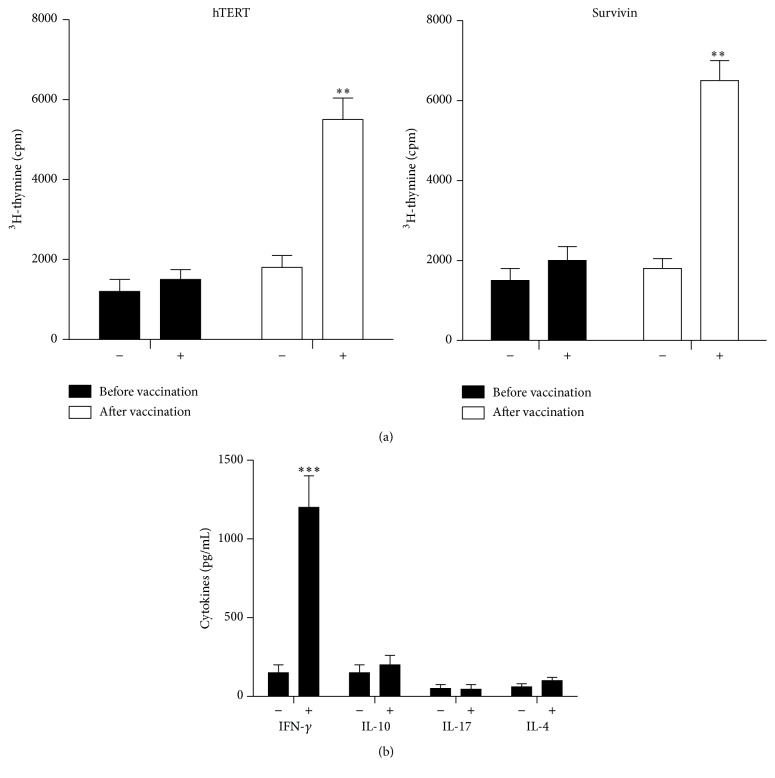
Analysis of T-cell responses to the vaccine. (a) T-cell responses to hTERT and survivin antigens prior to (week 0) and after (week 15) vaccination were analysed in PBMCs. The cells were not incubated (−) or were incubated (+) with recombinant hTERT or survivin protein (20 *μ*g/mL) for 6 days, and T-cell proliferation was determined by incorporation of [^3^H]-thymidine following overnight pulsing. The results are presented as the mean ± standard deviation of triplicate determinations. (b) Cytokine contents in culture supernatants. PBMCs were costimulated (+) or not (−) with recombinant survivin and hTERT (10 *μ*g/mL each) for 5 days and then cytokine contents in culture supernatants were analysed by commercially available ELISA kits. The results are presented as the mean ± standard deviation of triplicate determinations. The data are representative of two independent experiments. PBMCs: peripheral blood mononuclear cells; IL: interleukin; IFN: interferon. ^*∗∗*^ *P* < 0.02 and ^*∗∗∗*^ *P* < 0.001.

**Figure 3:**
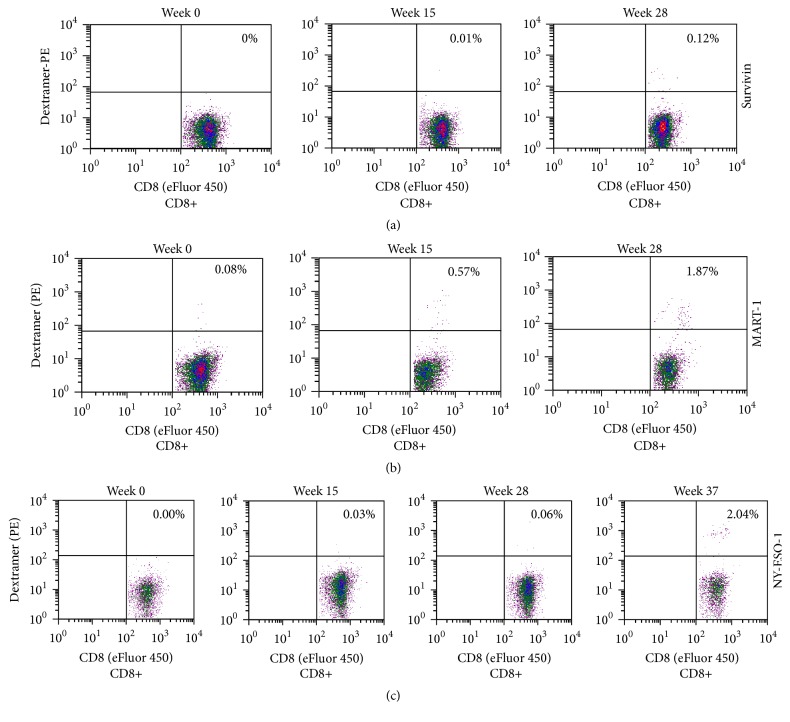
Analysis of CD8+ T-cell response following vaccination. PBMCs prior to (week 0) and following (weeks 15, 28 and 37) vaccination were cultured with either (a) HLA-A^*∗*^0201-restricted survivin, (b) MART-1, or (c) NY-ESO-1 peptide (10 *μ*g/mL). The cells were cultured in a 96-well plate, harvested at day 10, and then stained with phycoerythrin- (PE-) conjugated survivin (LTLGEFLKL), MART-1 (EAAGIGILTV), or NY-ESO-1 (SLLMWITQV) dextramer (Immudex) for 10 min followed by anti-CD8, anti-CD19, and anti-CD56 staining (20 min). Subsequent to being washed, gated CD8+ T-cell population was analysed for dextramer-staining on a BD SLR II flow cytometer.

**Figure 4:**
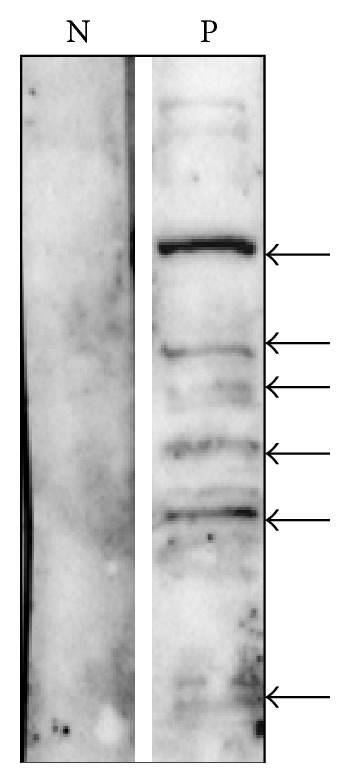
Protein extracts prepared from melanoma cell lines were separated by 10% SDS-PAGE (50 *μ*g/lane), transferred to nitrocellulose, and then incubated with patient serum (P) obtained at week 28 after immunisation. Immunoreactive proteins were detected using HRP-conjugated anti-human IgG. As a control, a serum from a normal donor (N) was used. Reactive proteins are indicated by the arrows.

Approximately three months after the vaccination, the patient developed antigen-specific responses to recombinant survivin and hTERT tumour antigens (Figure 2(a)). Cultured cells were also analysed for the expression of interferon-*γ*, IL-4, IL-10, and IL-17. A significant increase in interferon-*γ* expression after vaccination therapy was found (Figure 2(b)). The low levels of IL-4 indicated the presence of a Th-1 type response. Dextramer analysis revealed that the vaccine was able to expand the number of peripheral blood survivin-specific CD8+ T-cells (Figure 3(a), 0% versus 0.12%). Next, we assessed whether the patient developed an immune response against melanoma-associated Melan-A (MART-1) antigen (Figure 3(b)). A robust cytotoxic T-cell response was detected (0.08% versus 1.87%). We also analysed the response to NY-ESO-1, a cancer testis antigen that has been successfully targeted in vaccine and adaptive T-cell therapy trials in several solid tumours. Similarly, the patient developed a NY-ESO-1-cytotoxic T-cell response (Figure 3(c), 0% versus 2.04%). CD8+ T-cell response against NY-ESO-1 was not present prior to vaccination. In addition to T-cell responses, the patient developed antibody response to several melanoma proteins (Figure 4). It should be noted that antibody-induction by cancer vaccines may contribute to antitumour activity via antibody-dependent cellular cytotoxicity involving patient NK cells and macrophages.

### 2.3. Clinical Response

With respect to clinical response, one month after starting vaccination, the metastatic skin lesions significantly regressed and a mixed response was observed in the lung and liver, as assessed by computed tomography (CT). From then on, the patient achieved a significantly better performance status and experienced less pain. During the vaccine treatment, the patient continued to experience a remission in the skin lesions and a mixed response in the lung and liver metastatic lesions (Table 1). Subcutaneous metastasis was not detectable from February 2013. However, by the end of June 2013, the patient developed progression of the bone metastases. Palliative radiotherapy against the bone metastases was administered, and, on August 22, 2013, anti-PD1 treatment was initiated. After one cycle of treatment, the patient developed severe side-effects, and, due to progressive disease, no further therapy was administered. Unfortunately, the patient succumbed to the disease in October 2013.

## 3. Discussion

Therapeutic targeting of immune inhibitory molecules can boost immune responses to tumours and improve patient survival [18]. In the present study, a patient with advanced melanoma received ipilimumab therapy followed by an IDO-silenced DC vaccine. Immunological analysis in this patient revealed that the DC vaccine induced T-cell responses against the survivin and hTERT tumour antigens. The vaccine also increased the number of circulating MART-1- and NY-ESO-1-specific T-cells. This marked increase in the frequency of MART-1- and NY-ESO-1-specific CD8+ T-cells in the blood appeared later (weeks 28 and 37, resp.) after vaccination, supporting vaccine-induced epitope spreading. However, the involvement of anti-CTLA-4 therapy cannot be ruled out. In contrast to chemotherapy, the majority of the patients treated with ipilimumab often respond after 12 weeks from the beginning of the treatment [19, 20]. The present patient received IDO-silenced DC vaccine nearly 25 weeks after initial ipilimumab treatment. One month after vaccination, the patient achieved a stable disease state for duration of more than six months. Given the severity of the disease, this clinical observation is encouraging.

The activation of CD8+ T-cells against MART-1 and NY-ESO-1 in response to the vaccine and perhaps to ipilimumab illustrates how immunotherapies can diversify antitumour immunity. Moreover, the induction of B-cell response against several potential melanoma antigens some of which may be mutated proteins (neoantigens) suggests the presence of ongoing immune response that may explain the significant reduction in lung, liver, and skin metastases seen in this patient (Table 1). Although immune recognition of neoantigens has the potential to destroy developing tumours [21], in the present case resistance to therapy has occurred. Among the proposed mechanisms that contribute to tumour escape from the immune system attack is the genetic inactivation of the *β*2 microglobulin subunit of MHC class 1, which is relatively frequent in several tumour types, including melanomas [22]. Hence, in patients with impaired antigen presentation, the overexpression of tumour associated antigens and/or the appearance of neoantigens will not control tumour growth via TCR-activated T-cells. Thus, a vaccine concept based on neoantigens would require the analysis of antigen presentation pathways in tumour cells to determine their functionality. Moreover, IDO has been proposed as a potential mechanism to melanoma-derived immunosuppression [12]. In line with this notion, Allison and colleagues demonstrated that IDO can inhibit antitumour responses in the context of ipilimumab therapy by suppressing tumour-infiltrating effector T-cells, while favouring accumulation of Treg cells [23]. Therefore, there is an urgent need for combination therapies using siRNA technology or 1-methyl-tryptophan that is currently in Phase I clinical trials [24].

## Figures and Tables

**Table 1:** Analysis of metastases during vaccination.

Abdomen	Week 0	Week 15	Week 22	Week 26
Met A (Liver)	20	20	15	10
Met B (l.n.)	22	17	8	8
Met C (l.n.)	22	25	7	9
Met D (l.n.)	25	25	7	6
Met E (l.n.)	12	7	0	0

Sum	**101**	**94**	**37**	**33**

Met: metastasis, l.n.: lung node.
